# Dermatofibroma with Verocay Body-Type Palisading Features and a Brief Discussion on Potential Schwannoma Mimickers of the Skin

**DOI:** 10.5146/tjpath.2023.01602

**Published:** 2023-09-15

**Authors:** Yunus Baran Kok, Cuyan Demirkesen

**Affiliations:** Acibadem University, School of Medicine, Department of Pathology, Istanbul, Turkey

**Keywords:** Dermatofibroma, Palisading, Verocay

## Abstract

Several types of cutaneous tumors can show palisading features or the so-called rippled pattern. The list includes adnexal tumors such as trichoblastoma and sebaceoma, basal cell carcinoma, leiomyoma, perineuroma, myofibroblastoma, and even malignant melanoma. Dermatofibroma, which is known for having a large variety of histological patterns, is also in the list. Here we present a case of dermatofibroma with palisading features strikingly similar to Verocay bodies of schwannoma.

**Dear Editor,**

Dermatofibroma (cutaneous fibrous histiocytoma) is a common skin tumor well known by pathologists, although the wide varieties of its histological appearance can make it a mimicker of other, benign or malignant, skin tumors. Histopathological classification of dermatofibroma can be made by its architectural (deep penetrating, aneurysmal etc.) or cellular (clear cell, monster cell etc.) peculiarities, or both (1). One histological pattern can be prominent or two or more histological patterns can be combined in the same tumor (composite dermatofibromas). A palisading type is rarely seen (2 in 200 dermatofibromas in one study), and it includes other tumors in the differential diagnosis, also rarely presented on the skin, such as schwannoma (2). Here, we aim to present a case of this rare dermatofibroma variant and discuss cutaneous tumors with nuclear palisading features resembling Verocay bodies.

The patient was a 48-year-old male with no remarkable medical history. The dermatologist described widespread papular lesions on the chest and sent a punch biopsy with two clinical prediagnoses as eruptive dermatofibroma and histiocytosis. Microscopic sections revealed a neoplasm infiltrating the dermis and overlying a hyperkeratotic and hyperplastic epidermis (Figure 1). The neoplasm consisted of a diffuse proliferation of spindle and ovoid cells. The most superficial portion of the tumor was relatively cellular with a few multinucleated giant cells (Figure 1). The deep portion of the tumor showed a storiform pattern with short fascicles, collagenous stroma, fibrohistiocytic proliferation, and small clusters of foamy macrophages, giving this part of the tumor a more conventional dermatofibroma appearance (Figure 2). In between, there was a zone of palisading cells, indistinguishable from the Verocay bodies of schwannoma (Figure 2). The nuclei of these tumor cells were aligned in rows or palisades, and the cell processes were fused into eosinophilic masses, forming Verocay bodies. A brief immunohistochemical panel, consisting of anti-CD34, anti-Factor XIIIa, and anti-S-100, was performed to make the differential diagnosis between dermatofibroma and schwannoma, and to rule out dermatofibrosarcoma protuberans. In all compartments including Verocay body-like areas, the tumor cells showed immunoreactivity for Factor XIIIa while they were negative for CD34 and S-100 antibodies, and the latter two excluded DFSP and schwannoma, respectively (Figure 2). Therefore, the diagnosis was palisading dermatofibroma.

**Figure 1:**
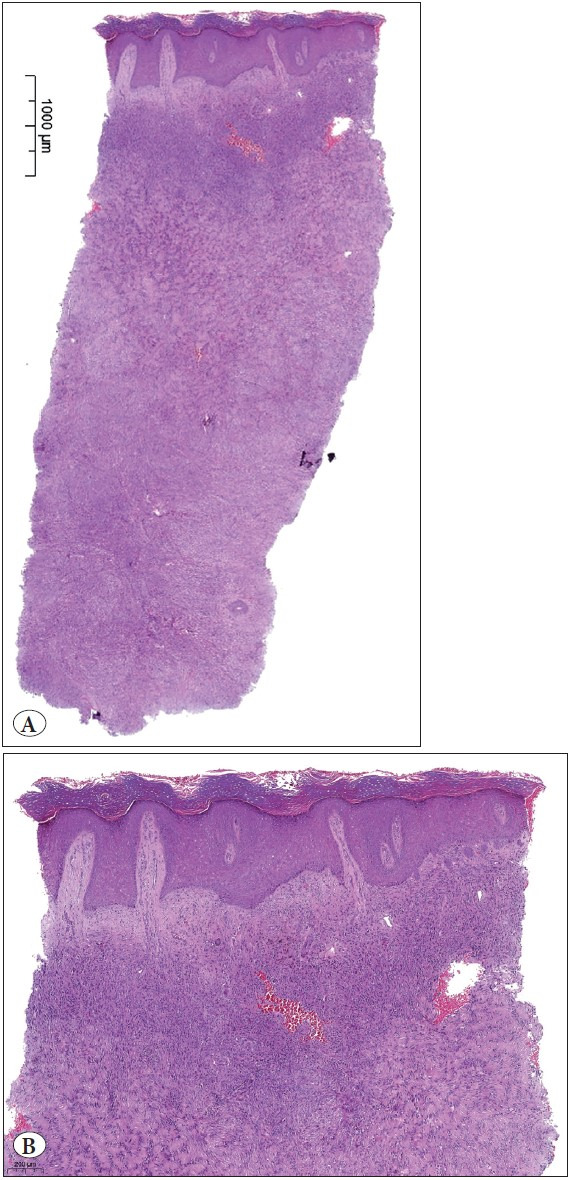
**A)** Neoplasm infiltrating dermis in a diffuse manner. At low magnification, a blend of different patterns can be seen. (H&E; x10). **B)** Upper part of the tumor. This part is cellular, composed of spindle and ovoid cells. Overlying epidermis shows hyperkeratotic and hyperplastic changes. (H&E; x40)

**Figure 2:**
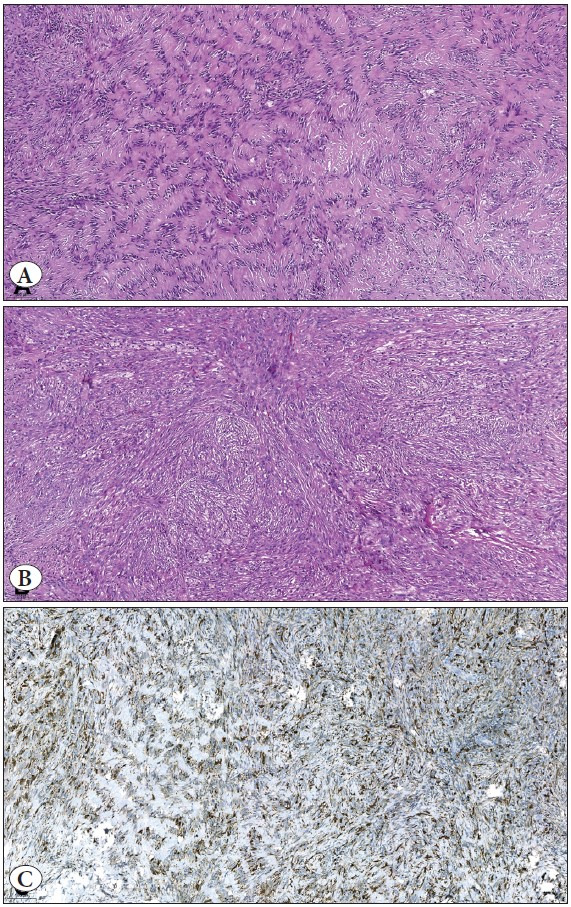
**A)** Middle part of the tumor with a striking Verocaybody like appearance. Acellular hyaline zones are lined with palisading elongated cells. This is a typical feature of schwannoma. (H&E; x100). **B)** Deeper part of the tumor. Fascicles of spindle cells, scattered inflammatory cells, and foamy macrophages are seen. These findings are typical for fibrohistiocytic tumors. (H&E; x100). **C)** Cytoplasmic Factor XIIIa expression of the tumor. Both Verocay body-like (left half of the figure) and fibrohistiocytic (right half of the figure) parts of the tumor are positive for the antibody. (IHC; x100).

Verocay body is described by rows of elongated palisading nuclei with alternating acellular zones. Laminin overexpression (2) and increased phospholipid deposition in the cellular matrix (3) are considered in the pathogenesis. Although it is thought to show neural differentiation and is usually identified in schwannoma, Verocay body-like palisading can be seen in other neoplasms of variable origin. Also referred to as a “rippled pattern” in certain skin tumors (4), Verocay body-like palisading is reported in trichoblastoma and sebaceoma, basal cell carcinoma, dermatofibroma and dermatofibrosarcoma protuberans, leiomyoma, palisaded encapsulated neuroma, perineuroma, myofibroblastoma, melanocytic nevus, and malignant melanoma (5). Basal cell carcinoma and adnexal tumors can be distinguished by their relationship with epidermal/adnexal structures and cytokeratin expression. Leiomyoma shows positivity for markers of muscle differentiation and no expression for S-100. Neural tumors are expected to be positive with S-100. Melanocytic tumors show positivity for melanocytic markers in addition to S-100 positivity. In our case, conventional fibrohistiocytic areas neighboring the Verocay body-like zone and the representative immunohistochemical profile made the diagnosis and no further investigation was required.

Palisading histology is a rare pattern of dermatofibroma. In two studies in which 200 dermatofibromas in one and 122 dermatofibromas in the other were examined histopathologically, 2 of each were found to be the palisading type (2% and 1.6% respectively) (6,7).

In conclusion, it is important to keep in mind that dermatofibroma is one of the tumors that may form Verocay bodies.

## Conflict of Interest

The authors declare no conflicts of interest.
